# Dogs Rely On Visual Cues Rather Than On Effector-Specific Movement Representations to Predict Human Action Targets

**DOI:** 10.1162/opmi_a_00096

**Published:** 2023-08-20

**Authors:** Lucrezia Lonardo, Christoph J. Völter, Claus Lamm, Ludwig Huber

**Affiliations:** Comparative Cognition, Messerli Research Institute, University of Veterinary Medicine of Vienna, Medical University of Vienna and University of Vienna, Vienna, Austria; Social, Cognitive and Affective Neuroscience Unit, Department of Cognition, Emotion and Methods in Psychology, Faculty of Psychology, University of Vienna, Vienna, Austria

**Keywords:** social cognition, action prediction, canine cognition, anticipatory looks, eye-tracking

## Abstract

The ability to predict others’ actions is one of the main pillars of social cognition. We investigated the processes underlying this ability by pitting motor representations of the observed movements against visual familiarity. In two pre-registered eye-tracking experiments, we measured the gaze arrival times of 16 dogs (*Canis familiaris*) who observed videos of a human or a conspecific executing the same goal-directed actions. On the first trial, when the human agent performed human-typical movements outside dogs’ specific motor repertoire, dogs’ gaze arrived at the target object anticipatorily (i.e., before the human touched the target object). When the agent was a conspecific, dogs’ gaze arrived to the target object reactively (i.e., upon or after touch). When the human agent performed unusual movements more closely related to the dogs’ motor possibilities (e.g., crawling instead of walking), dogs’ gaze arrival times were intermediate between the other two conditions. In a replication experiment, with slightly different stimuli, dogs’ looks to the target object were neither significantly predictive nor reactive, irrespective of the agent. However, when including looks at the target object that were not preceded by looks to the agents, on average dogs looked anticipatorily and sooner at the human agent’s action target than at the conspecific’s. Looking times and pupil size analyses suggest that the dogs’ attention was captured more by the dog agent. These results suggest that visual familiarity with the observed action and saliency of the agent had a stronger influence on the dogs’ looking behaviour than effector-specific movement representations in anticipating action targets.

## INTRODUCTION

Humans and non-human primates can visually predict the target object of others’ goal-directed actions (Flanagan & Johansson, 2003; Myowa-Yamakoshi et al., 2012). To disentangle the contribution to this ability of the observer’s motor experience from that of the observer’s visual experience with the observed movements, we tested dogs (*Canis familiaris*), a species whose social cognition and behaviour are deemed functionally comparable to ours in many aspects (Hare & Tomasello, 2005; Topál et al., 2009). Crucially, unlike primates, dogs lack motor experience with certain human actions but, unlike human infants, adult dogs have extensive visual experience with human actions and a fully developed motor system. For these reasons, they provide a compelling opportunity to assess the relative importance of motor simulation and visually-driven processes, two of the cognitive components that supposedly subtend action prediction.

Hypotheses about the development and nature of the processes underlying action prediction in humans vary considerably (Biro & Leslie, 2007; Brass et al., 2007; Csibra, 2008; Sommerville et al., 2012; Van Overwalle & Baetens, 2009). On the one hand, our capacity to infer others’ goals (the immediate targets of their actions) could be based on a “direct matching”, whereby the movements and goals of an observed agent are directly matched with the observer’s corresponding movement representations (Rizzolatti et al., 2001; Rizzolatti & Sinigaglia, 2010). This mechanism would be supported by neural processes that would allow to simulate others’ actions using the observer’s own motor system (Rizzolatti et al., 2014). The simulation account postulates that organisms activate their own motor plans for the observed movements in order to replicate internally what others are doing when executing those movements—and hence identify their goal (Gallese et al., 2004). A similar view has been embraced by developmental psychologists who indeed showed the importance of first-person motor experience in gaining insights about others’ action targets (Sommerville et al., 2012; Woodward et al., 2009).

On the other hand, the presence of specific behavioural cues (such as equifinal movement towards a target, efficiency of movement, self-propulsion, contingent reactivity) appears sufficient to trigger the perception of agency and goal-directedness in humans (Biro & Leslie, 2007; Csibra et al., 2003; Gergely et al., 1995; Heider & Simmel, 1944; Johnson et al., 1998; Premack, 1990), but not in monkeys (Schafroth et al., 2021), independently of how similar the observed agent and actions are to the observer’s own motor representations. Moreover, the discovery that goal saliency and certainty (unrelated to the observer’s motor system) influence goal-based predictive gaze shifts (Adam & Elsner, 2020; Eshuis et al., 2009; Henrichs et al., 2014) supports the notion that action prediction might also rely on a form of inferential reasoning (such as “emulative action reconstruction”; Csibra, 2008). In contrast to the simulation account, the emulative account assumes that motor activation in response to the observation of others’ actions follows (rather than enables) the identification of others’ goals and reflects the observers’ reconstruction of how to use their own motor system to achieve the same goal (Csibra & Gergely, 2007). Finding that dogs do not base their prediction on a direct matching process might indicate that visual familiarity with the observed action plays a more important role in action prediction than commonly assumed and that, in addition to a direct motor matching, other processes, such as emulative action reconstruction, might be at the basis of action prediction.

Pet dogs (*Canis familiaris*) provide an excellent opportunity to test the direct matching hypothesis. Developmental studies with human infants have highlighted the influence of first-person action production on the ability to identify the target of those actions when executed by others (Krogh-Jespersen & Woodward, 2018; Sommerville et al., 2008). Unlike the (primate) species tested so far, due to their different anatomy and locomotion, dogs do not perform (and likely lack effector-specific motor representations of) human movements such as bipedal walking and grasping with fingers. At the same time, however, over the course of their ontogeny, pet dogs gain extensive visual experience with human goal-directed actions. Therefore, studying dogs can help disentangle the role of visual familiarity from that of motor representations acquired via self-produced actions. Secondly, unlike infants, adult dogs exhibit a fully developed motor repertoire and hence can help elucidate the specificity of action prediction during the course of human development. That is, whether younger infants’ lack of action prediction depends on the impossibility to execute the observed, specific, motor patterns or rather on a generally immature motor system (Southgate, 2013). Moreover, previous studies provided indication that dogs pay attention to human goal-directed actions. Dogs proved to be able to reproduce with their own behaviour the observed goal-directed actions of a human demonstrator, even after some delay. The dogs spontaneously employed actions within their motor repertoire to imitate the actions demonstrated by a human (Fugazza & Miklósi, 2014; Huber et al., 2009; Topál et al., 2006). They preferred to emulate a demonstrated action when its target object was clearly identifiable and to imitate the movements when the action appeared non goal-directed (Fugazza et al., 2019). Further, their imitative and emulative responses seem to reflect a consideration of the demonstrator’s constraints when performing the action (Range et al., 2007; although see also Kaminski et al., 2011; Huber et al., 2012). Finally, dogs can be trained for accurate static eye-tracking (Karl et al., 2020), which allows for measuring visual attention in a precise, objective, and comparable way to research on human infants and nonhuman primates.

With the present study we started at a basic level, by asking whether dogs are able to visually anticipate the immediate target of others’ actions in a simplified scenario, with only one, visually salient, target object, similarly to previous studies (e.g., Falck-Ytter et al., 2006). The ability to infer the target of observed actions as they unfold has been investigated by measuring online visual prediction. Specifically, researchers have made use of eye-tracking technology to measure in real time the participants’ gaze arrival times to an action target relative to the moment in which an observed agent reaches it. Developmental and comparative researchers have then typically correlated (more or less directly) this measure with the participants’ motor skill to perform the observed action, to investigate the link between action prediction and production. In accordance with the direct matching hypothesis, human adults exhibit goal-based anticipatory looks (i.e., looks to the target happening before this is actually reached by the agent) not only when directly executing a goal-directed action but also when observing others execute the same action (Flanagan & Johansson, 2003). Many subsequent studies consistently found an association between the observers’ motor experience performing a certain action and their ability to visually anticipate its outcome when observing others execute it (Ambrosini et al., 2013; Brandone, 2015; Cannon et al., 2012; Falck-Ytter et al., 2006; Gredebäck & Melinder, 2010; Kanakogi & Itakura, 2011; Kochukhova & Gredebäck, 2010; Krogh-Jespersen & Woodward, 2018; Myowa-Yamakoshi et al., 2012; Stapel et al., 2016). For example, Myowa-Yamakoshi et al. (2012) showed videos of an actress pouring juice into a glass to participants of different age groups: adults, 12-month old and 8-month old infants. While the adults were capable of performing the action shown in the video, the 12-month olds could only perform a simplified version of it and the 8-month olds were incapable of performing the action at all. Consistently with the direct-matching hypothesis, only the adults looked at the glass before the onset of pouring, thus anticipating the target of the action. The 12-month olds did not gaze at the glass predictively but rather at the same time as the onset of pouring and the 8-month olds looked at the glass reactively, that is, only after the onset of pouring. The authors showed the same video to a group of captive chimpanzees (*Pan troglodytes*) as well, who have been observed pouring liquids from one container to another in their enclosure. Interestingly, chimpanzees anticipated the human’s action target similarly to human adults, by landing with their gaze on the glass approximately 700 ms before the onset of pouring. Because the chimpanzees likely had movement representations of the action whose outcome they could predict, these results were considered as evidence in favor of the direct matching hypothesis.

However, a direct matching mechanism based on the observer’s own representation of the observed action might not be necessary for action prediction and understanding. Other mechanisms as well appear to subtend this ability, as evidenced by studies of observers lacking motor representations of the observed actions. For example, an EEG study found that 9-month old infants recruit their sensorimotor cortex when observing impossible actions, i.e., actions that the human body is not capable of performing, such as bending the arm and elbow in biomechanically unfeasible ways (Southgate et al., 2008). Southgate (2013) proposed that some of the data from infants’ anticipatory looking paradigms and adults’ TMS studies (e.g., Elsner et al., 2013) are compatible with an alternative explanation relative to the direct matching hypothesis. For example, according to the emulative action reconstruction account (Csibra, 2008), the activation of the observer’s motor system would be a consequence (rather than the cause) of target identification and it would reflect the observer’s top–down processing of the means used to obtain the goal (Southgate, 2013).

In line with previous research (e.g., Brandone et al., 2014; Falck-Ytter et al., 2006; Myowa-Yamakoshi et al., 2012), we measured gaze arrival times (i.e., the moment in which the dogs shifted their gaze to the target for the first time) and operationalized action prediction as looks to the target object before the agent made contact with it. If the ability to predict others’ actions rests upon a direct matching mechanism, as it has been suggested for primates (Rizzolatti et al., 2001; Rizzolatti & Sinigaglia, 2010), we predict that dogs would look at the action target sooner when the agent is a dog rather than a human. We expected dogs to anticipate the action target mainly when the agent was a dog. Indeed, dogs should possess effector-specific movement representations, derived from first-person experience, of the actions executed by a conspecific. In contrast, the lack of movement representations of the actions executed by a human would make it difficult for the dogs to make sense of such actions. Conversely, dogs’ anticipatory looks to the target object acted upon by heterospecifcs (humans) would be better explained by emulative action reconstruction (Csibra, 2008).

To test the relative contribution to online action prediction of visual familiarity and motor experience with the observed movements, we contrasted conditions in which either a human or a dog executed the same goal-directed action. To tease apart the effect of the agent’s species from that of the movements employed to reach the goal, we contrasted conditions in which the dog agent executed a goal-directed action versus a human agent executed the same goal-directed action but performed in a dog-like manner. To test whether visual familiarity with an action influences the prediction of its outcome, we contrasted the conditions in which the human agent executed a goal-directed action in a human-like versus a dog-like manner, since dogs are likely more unfamiliar with humans moving in the latter way.

In detail, the dogs were tested in two experiments with three identical conditions but differing stimuli sets. In the first experiment, the goal-directed action was the displacing of a ball, while in the second experiment it was the lifting of a stuffed toy animal. We chose to show, across the two experiments, two different actions, performed using two different effectors, in order to assess how dependent on the specific action and effector the results were. In choosing which actions to show, we reasoned that dogs were probably familiar with the ordinary human actions of kicking a ball and picking up a toy. We tried to have the conditions with the dog actor replicate functionally the human actions. Hence, in Experiment 1, the dog uses a limb and its extremity to push the ball forward and in Experiment 2 she grasps the toy using her mouth. Finally, because we were interested in all pairwise comparisons between conditions, within the same experiment the conditions in which the human moves more similarly to a dog and the conditions with the dog actor needed to show the same effector being used.

Moreover, Experiment 2 was conducted to assess the robustness of the measures when the dogs were tested again on similar stimuli, given that anticipatory looks in infants do not always seem to be a replicable measure and might depend on subtle contextual factors that have not yet been clearly identified (Margoni et al., 2022, for an overview).

We not only measured the dogs’ gaze arrival times to the target objects, but we also explored their looking times to the agents’ faces and bodies and their pupil sizes. The gaze arrival times served to assess whether the dogs looked to the target of the action before the agent made contact with it. We measured the dogs’ looking times to the agents (i.e., how long their gaze was detected within the agents’ areas of interest) to assess which species (conspecific or human) and parts of the agents (face or body) were more salient. In addition, we measured the dogs’ pupil sizes as correlates of the dogs’ attention level toward the depicted scene. If dogs have expectations about common human actions, derived from their extensive visual experience with them, we expected longer looking times and increased pupil size (both potential indicators of greater surprise) for the human agent moving in a dog-like manner than for the human agent moving normally.

## METHODS

Experimental design, hypothesis, predictions, sample size and size of the target areas of interest (AOIs), for the gaze arrival time analyses, were pre-registered: https://osf.io/8akvu.

### Subjects

The same 16 dogs (6 females) of various breeds participated in both experiments. Table S1 provides demographic and procedural information. The tested dogs’ average age at the beginning of testing was 52 months (range: 20–139).

### Design

In both experiments, we tested the dogs in three conditions: (1) a dog performing the goal-directed action (*dog* conditions), (2) a human performing the same action while making movements outside of dogs’ motor possibilities (*human outside* conditions), and (3) the same human performing the same action by making movements closer to, or within, the dogs’ motor possibilities (*human within* conditions). Each dog was presented with four trials (identical video repetitions) of one condition on each of three different testing days, usually separated by one week. The order of presentation of the conditions was counterbalanced across dogs with regards to sex and age. On a testing day, dogs participated in up to three experiments. In almost all of the sessions, the two experiments reported in this study were presented on the same day (with the exception of two sessions of two dogs, whereby only one of the two experiments was shown). In any case, Experiment 1 always preceded Experiment 2.

### Stimuli

Each video (see supplementary materials) had a frame rate of 100 frames per s, a resolution of 1024 × 768 pixels and lasted 8 s (including the extensions described below). The videos were shown on a 24-inch LCD monitor with refresh rate of 100 Hz, positioned 70 cm away from the dogs’ eyes.

For Experiment 1, the video of the *dog* condition showed a mixed-breed female dog (of approximately the same size as the subjects) look at and approach a static yellow ball before pushing it away using her left front paw. The videos with a human agent showed a female experimenter look at, approach and push the same ball away. In the condition *human outside*, the agent walked and kicked the ball away using her left foot; in the condition *human within*, the agent crawled and pushed the ball away using the back of her left hand. The initial pose (crouching/sitting, facing the camera) of the human and dog agents was the same in all conditions. In all three conditions, the ball rolled out of the scene and the agent continued moving in the same direction and with the same movements as during the approach phase.

In Experiment 2, a conceptual replication of Experiment 1, the same actors and scene were kept, while the target object was replaced by a blue stuffed elephant. The video of the *dog* condition showed the dog looking at, approaching and grasping with her mouth the elephant. The video of the *human outside* condition showed the experimenter looking at, walking to and grasping with her left hand the elephant. The video of the *human within* condition showed the experimenter looking at, crawling to and grasping with her mouth the elephant. Again, the initial pose of the dog and human agent was the same in all conditions. In all three conditions, the elephant was lifted and carried outside of the scene by the agents, who left the scene as well; therefore, the last 2 s of the videos showed just the empty room.

In both experiments, at the beginning of the videos the agents (equally unfamiliar to the subjects) faced the camera. In all three conditions, the agent’s starting position was on the right side of the screen, while the target object was positioned on the left side of the screen. The first frame of each of the videos, in which the agents were looking directly into the camera, was presented statically for 1 s before the rest of the video was presented at real-life speed. This ensured that the dogs had sufficient time to explore the scene freely, before the agents started to move. Approximately 2 s after the beginning of the video, the actor started to orient with the head and body towards the goal object and to approach it (2 s). In each of the two experiments, the three videos were synchronized with regard to the moment in which the agents entered the target AOI. The last frame of each of the three videos was frozen for 2 s.

Within each experiment, the static target AOI was placed in the same coordinates across the three videos.

### Procedure

Before taking part in the experiments, the dogs were trained to place their head on a chinrest, irrespective of the whereabouts of their trainer, and to perform a calibration and subsequent validation of the position of their fixations on the screen. The criterion for considering the training phase concluded successfully was an average difference between calibration and validation smaller than 1° of visual angle. For details about the training see Karl et al. (2020).

The heights of the chinrest and eye-tracker (EyeLink 1000; SR Research, Canada) were adjusted to the size of each subject at the beginning of each session. During the whole session, water was available to the dogs. Interested owners were allowed to remain in the room during the experiments and watch the screen from behind their dog’s back, at a distance of approximately two meters from the dog. Before each session, each subject performed a 5-point calibration (with the first point repeated at the end). The size of the calibration targets ranged from 24 × 24 px to 64 × 64 px (the ideal size of the calibration target for each individual was determined by the dog trainer during the training phase). The dogs’ right eye was tracked at 1000 Hz.

At the beginning of each trial (i.e., video presentation), an animation was presented centrally. Only if dogs fixated it for at least 50 ms, was the first frame of the video presented. Therefore, at the beginning of each trial, the dogs’ gaze was centered between the agent and the target object. After each video presentation, a grey screen would appear until the following fixation animation or until the end of the experiment. If a trial had to be terminated before the end of the video, for example due to the dogs leaving the chinrest, the same trial, and, if applicable, the following ones, were repeated, after a new calibration, during the same session or on a following one.

### Statistical Analyses

In both experiments, we measured dogs’ gaze arrival times to the target AOI (164 × 642 pixels), their looking times to the agents’ face and body and dogs’ pupil size over the course of the trial. The data from the two experiments were analysed separately but following the same steps, using the software R (R Core Team, 2022), version 4.1.2. *P*-values smaller than 0.05 were used as criterion for significance testing.

#### Probability to Look at the Target AOI.

In a minority of trials, the dogs did not look at the target object. We fitted a binomial GLMM (one per experiment; Baayen, 2008) to check whether the probability that dogs looked at the target at least once during a trial differed significantly between conditions. For Experiment 1, we included as only test predictor the condition and as control predictors the trial number and the order in which the conditions were presented. As random effects, we included the random slope of condition and trial number within subject. Finally, the correlations between random slopes and intercept were included as well. For Experiment 2, in order for the model described above to converge, we had to remove both random slopes and only leave the random intercept of subject.

#### Gaze Arrival Times Into the Target AOI.

We measured the dogs’ standardised gaze arrival times: the gaze arrival times into the target AOI relative to the mean time when the agents in the videos entered the target AOI. The latter was defined as the last millisecond in which the frame preceding the moment in which the agent entered the target AOI was shown in each trial. Because we subtracted the mean agents’ arrival time from the dogs’ gaze arrival time, negative values of standardised gaze arrival time are indicative of predictive looks.

For both experiments, we transformed the gaze arrival times into the proportion of trial time elapsed before dogs looked into the target AOI after their first look to the agent and we fitted to this response variable GLMMs (one for each experiment; Baayen, 2008) with beta error structure and logit link function (Bolker, 2008; McCullagh & Nelder, 1989). We included condition, trial number and order of presentation of the conditions as fixed effects and the random slopes of condition, order of conditions and trial number within subject.

Similar models (one for each experiment) were fitted to analyse the first trial separately. The only differences relative to the models analysing all trials were the exclusion of the random slope of condition and of order of conditions (not identifiable) and of the fixed and random effect of trial number. For Experiment 1, a beta model was used for consistency with the rest of the gaze arrival time analyses, although an equivalent LMM could have been employed (residuals normally distributed).

We excluded from these analyses trials in which dogs never directed their gaze to the target AOI during the duration of the whole video.

As pre-registered, we only included trials in which dogs looked at least once at the agent before looking at the target and, within these trials, we only considered the looks into the target AOI that happened after the first look at the agent. For Experiment 2, we additionally explored the arrival times into the target AOI, considering all trials in which dogs looked at the target AOI, irrespective of whether they had previously gazed into the agent AOI or not. We fitted to the proportion of trial time elapsed before dogs’ gaze sample was detected in the target AOI GLMMs (one for all trials, one only for the first trial) with beta error structure and logit link function, identical to the ones described above.

For both experiments, two-tailed *t*-tests were used to assess whether the average standardised gaze arrival times (relative to the mean agent arrival time) in the three conditions were significantly different from 0 (i.e., predictive or reactive relative to the agents’ actions), both aggregating all trials per condition and considering only the first trial per condition.

#### Looking Times to the Agents.

For both experiments, we analysed both the absolute and standardized looking times to the agents’ adjacent face and body AOIs. The standardized looking times were obtained by dividing the absolute looking time into the agent’s dynamic AOIs (face or body) by the size (pixels) of that AOI. When a gaze sample happened to fall on the border between the face and the rest of the body AOIs, the looking time was assigned to both AOIs.

For each experiment, to analyse the effect of condition (*dog*/*human within*/*human outside*), AOI (agent’s face/body) and their interaction (fixed effects) on our subjects’ standardised looking times to the agents, we fitted a GLMM with beta error structure and logit link function. We included in this full model the interaction between the two test predictors because dogs’ might have allocated their attention to the moving agents’ face or body differently across agents’ species (conspecific vs. heterospecific) and type of movements (within or outside their own motor repertoire). Additional fixed effects were the trial number (1 to 4) and the order of conditions (1 to 3).

#### Pupil Size.

For both experiments, we measured the pupil size (pixel count of the area occupied by the pupil) throughout the video presentation. Data were pre-processed as described in the supplementary materials, according to the recommendations in Fink et al. (2023) and Mathôt et al. (2018).

For each experiment, the preprocessed and down-sampled (to 10 Hz) data were analyzed, as described by van Rij et al. (2019), with a generalized additive mixed model (GAMM) with Gaussian error structure, fitted using the function “bam” of package “mgcv” (Wood, 2011), with smoothing parameter selection method set to “ML”. We included a linear term for condition and smooth terms for time and for the interaction between time and condition, both with maximum number of knots set to 20. We included the non-parametric interaction between time and condition to account for a possible nonlinear effect of condition over time. We additionally included a smooth term for the interaction between X and Y gaze coordinates, as the gaze position on the screen might have influenced pupil size (Mathôt, 2018). Finally, we included a random factor smooth for each combination of subject, trial number and condition (event).

Additional details for all analyses and results are reported in the supplementary materials.

## RESULTS

### Probability to Look at the Target AOI

#### Experiment 1.

The dogs never looked at the target in two trials of the *dog* condition, in 13 trials of the *human outside* condition and in five trials in the *human within* condition. Hence, in Experiment 1, the probability that dogs looked at the target at least once during a trial was not influenced by condition (*χ*^2^ = 1.67, *df* = 2, *P* = .433). However, dogs were less likely to look at the target with increasing trial number (*χ*^2^ = 4.27, *df* = 1, *P* = 0.039).

#### Experiment 2.

In Experiment 2, the dogs did not look at the target in seven trials of the *dog* condition, in nine trials of the *human outside* condition and in one trial of the *human within* condition. The probability of the dogs to look at the target in the second experiment was influenced by both condition (*χ*^2^ = 14.09, *df* = 2, *P* = .001) and trial number (*χ*^2^ = 9.75, *df* = 1, *P* = .002), again with dogs being less likely to look at the target with increasing trial number. The dogs were less likely to look at the target in the *dog* than in the *human within* condition (Wald test: *z* = 2.42, *P* = .016) and less likely to look at the target in the *human outside* than in the *human within* condition (*z* = 2.66, *P* = .008).

### Gaze Arrival Times Into the Target AOI

#### Experiment 1.

**Figure 1.**
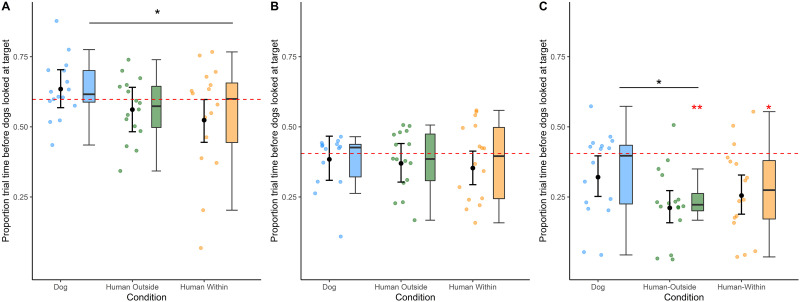
The boxplots show the distribution across all trials of the gaze arrival times into the target AOI in the three conditions of Experiments 1 (A) and 2 (B and C). Panel C shows the gaze arrival times to the target object AOI including the looks that were not preceded by a look to the agent AOI. The points show each individual’s mean standardised arrival time to the target AOI across all four trials. The red dashed line corresponds to the moment in which the agents entered the target AOI in the video. Negative values on the *y*-axis indicate predictive looks relative to the agent’s entering the target AOI. The red asterisks indicate significantly predictive gaze arrival times (one sample *t*-test). The black asterisks show a significant difference between conditions (beta GLMM). The black dots with error bars show the fitted values and confidence intervals of the beta models.

Condition had a significant influence on dogs’ gaze arrival times (*χ*^2^ = 8.06, *df* = 2, *P* = .018; for more details on the beta GLMM see Table S2). Dogs looked at the target AOI sooner in the *human within* than in the *dog* condition (Figure 1A, Wald test: *z* = −2.68, *P* = .008) but only by trend sooner in the *human outside* than in the *dog* condition (*z* = −1.88, *P* = .060). There was no difference in the gaze arrival times between the two human conditions (*z* = .99, *P* = .320). The control predictors (trial number and order of conditions) had no significant effect.

On average across all trials, dogs’ gaze arrival times into the target AOI were neither significantly predictive nor significantly reactive in any of the three conditions.

**Figure 2.**
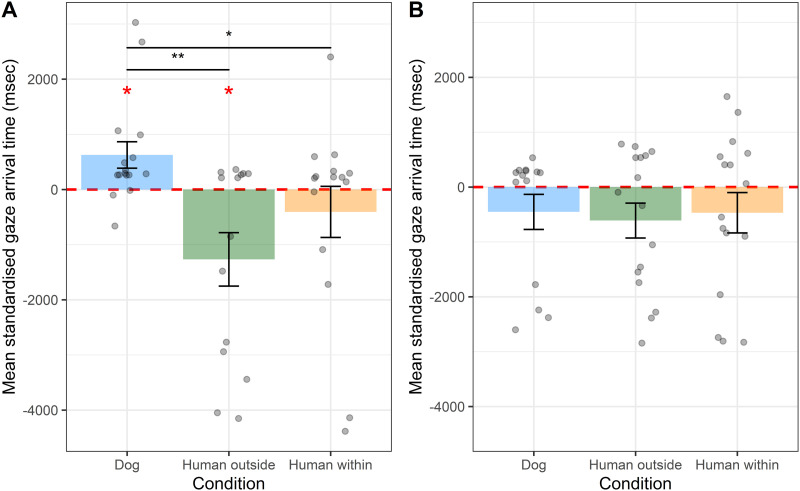
Mean standardised gaze arrival times in the first trial of Experiment 1 (A) and 2 (B). Error bars represent the standard error. The points show each individual’s standardised arrival time to the target AOI. The value of zero on the *y*-axis corresponds to the moment in which the agents entered the target IA. Negative values indicate predictive looks relative to the agent’s entering the target AOI. The black asterisks indicate a significant difference between conditions (beta GLMM; * *p* < .05; ** *p* < .01). The red asterisks indicate a significant difference from zero (one sample *t*-test).

The results of the beta GLMM fitted to the data of the first trial are summarised in Table S3. Already in the first trial, dogs’ gaze arrival times into the target AOI were modulated significantly by condition (*χ*^2^ = 14.17, *df* = 2, *P* = .001; Figure 2A). Specifically, on average dogs looked at the target of the action 627 ms after the dog agent had entered the target AOI. Hence, their gaze was significantly reactive in the *dog* condition (*t* = 2.619, *df* = 15, *P* = .019). Instead, the average standardized gaze arrival time in the first trial of the *human outside* condition was −1267 ms, which was significantly predictive (*t* = −2.61, *df* = 13, *P* = .021). In the *human within* condition, dogs’ average gaze arrival times were intermediate between the two other conditions (−406 ms) and were neither significantly predictive nor reactive (*t* = −0.88, *df* = 14, *P* = .395). On the first trial, the average standardized gaze arrival times were significantly smaller in both the *human outside* (*z* = −4.03, *P* < .001) and *human within* (*z* = −2.59, *P* = .01) conditions than in the *dog* condition. The gaze arrival times did not differ significantly between the two human conditions (*human outside* − *human within*: *z* = −1.57, *P* = .117).

#### Experiment 2.

As shown in Figure 1B, overall, there was no effect of condition on dogs’ gaze arrival times (see Table S4). With increasing trial number, the dogs took significantly longer to look at the target (*χ*^2^ = 7.86, *df* = 1, *P* = .005). The order of presentation of the conditions did not have a significant influence on dogs’ gaze arrival times into the target AOI.

In the first trial per condition, dogs’ gaze arrival times were not modulated by condition nor order of presentation of the conditions (Table S5; Figure 2B). On average across all trials, dogs’ gaze arrival times into the target AOI were neither significantly predictive nor significantly reactive in any of the three conditions. In the first trial, dogs’ gaze arrival times into the target AOI were not significantly predictive or reactive in any of the three conditions.

Given that the dogs in Experiment 2 already had experience (gained in Experiment 1) with the overall scene involving a human and dog agent approaching a target object, we next conducted an exploratory analysis including all trials in which dogs looked at the target AOI (even those in which looks to the target were not preceded by looks to the agent). Overall, dogs’ gaze arrival times to the target were significantly modulated by condition (*χ*^2^ = 9.44, *df* = 2, *P* = .009; Table S6 and Figure 1C) but not from the first trial (Table S7). Indeed, on the first trial, dogs’ gaze arrived at the target object predictively in all three conditions (*dog*: *t* = −2.72, *df* = 14, *P* = .017; *human outside*: *t* = −4.82, *df* = 15, *P* < 0.001; *human within*: *t* = −3.41, *df* = 15, *P* = .004). On the first trial, there were no significant differences in the gaze arrival times between conditions (*human outside* − *human within*: *z* = −.34, *P* = .735; *human outside* − *dog*: *z* = −1.10, *P* = .274; *human within* − *dog*: *z* = −.76, *P* = .445).

Across trials, dogs’ gaze arrived into the target AOI significantly predictively when the agent was a human (*human outside*: *t* = −5.60, *df* = 15, *P* < 0.001; *human within*: *t* = −3.27, *df* = 15, *P* = .005) but not when it was a dog (*t* = −1.94, *df* = 15, *P* = .072). On average, dogs looked sooner at the target in the *human outside* condition than in the *dog* condition (*z* = −3.64, *P* < 0.001). Their average gaze arrival time did not differ between the *human within* and the *human outside* condition (*z* = −1.04, *P* = .297) nor between the *human within* and the *dog* condition (*z* = −1.860, *P* = .063).

### Looking Times to the Agents

#### Experiment 1.

When analysing the standardised looking times, the full model explained the results better than the null one (*χ*^2^ = 36.59, *df* = 3, *p* < 0.001). As shown in Table S8, we found a significant main effect of condition on dogs’ looking times (*χ*^2^ = 29.74, *df* = 2, *p* < 0.001). In particular, dogs looked longer at their conspecific than at the human agent moving similarly to a dog (*dog* − *human within*: *t* = 3.18, *P* = .005) and longer than at the human agent moving normally (*dog* − *human outside*: *t* = 7.70, *P* < .001). They also looked longer at the human moving similarly to a dog than at the human moving normally (*human outside* − *human within*: *t* = −3.60, *P* = .001). We also found a significant main effect of AOI (Figure S1), with dogs looking longer at the agents’ faces than bodies, irrespective of condition (*χ*^2^ = 6.87, *df* = 1, *P* = .009). Finally, dogs looked at the agents for shorter periods of time as the trial number increased (*χ*^2^ = 8.06, *df* = 1, *P* = .005).

#### Experiment 2.

When analyzing the standardized looking times (Figure S2), the full model explained the results significantly better than the null one (*χ*^2^ = 52.65, *df* = 5, *P* < .001; Table S9). The interaction between condition and AOI was significant (*χ*^2^ = 8.30, *df* = 2, *P* = .016). Additionally, dogs looked less at the agent with increasing trial number (*χ*^2^ = 8.84, *df* = 1, *P* = .003). Pairwise comparisons revealed that dogs looked significantly longer at their conspecific’s face than body (body − face: *t* = −3.79, *P* = .002). They also looked longer at their conspecific’s face than at the human’s face (*dog* face − *human outside* face: *t* = 5.67, *P* < .001; *dog* face − *human within* face: *t* = 4.05, *P* = .001) and body (*dog* face − *human outside* body: *t* = 4.77, *P* < .001; *dog* face − *human within* body: *t* = 4.78 < 0.001).

### Pupil Size

#### Experiment 1.

**Figure 3.**
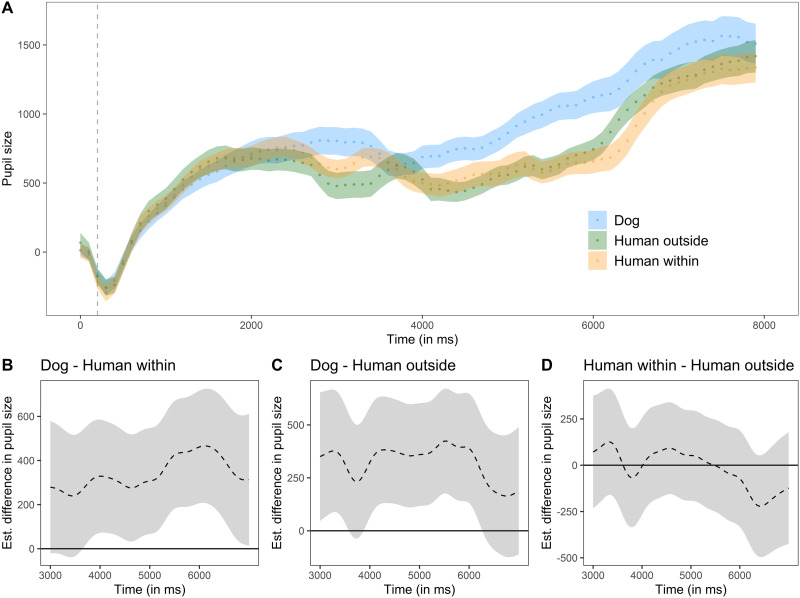
Experiment 1: A) time course of dogs’ average pupil size (arb. unit), baseline corrected and down-sampled, over the duration of the whole trial. The grey dashed line indicates the end of the baseline period. The coloured dotted lines show, for each condition, the average pupil size (each dot corresponds to the average of each bin) ± standard error. B), C) and D) difference curves between conditions based on GAMM predictions. Data are plotted as estimated difference in pupil size (dashed black line) ± 95% confidence intervals (grey area).

The pre-processed pupil size across the three conditions is plotted in Figure 3A. The full model explained the results significantly better than a null model lacking the condition factor and the non-parametric regression lines of the condition levels over time (*χ*^2^ = 48.21, *df* = 8, *p* < 0.001; AIC difference: 121.57; Table S10). Dogs exhibited a larger pupil size when watching the conspecific than the human agent (comparison *human outside* − *dog*: *t* = −2.81, *P* = .005; comparison *human within* − *dog*: *t* = −2.99, *P* = .003). The difference curves (Figure 3B–D) show that there was no significant difference in pupil size between the two conditions in which the agent was human. In these two conditions, but not in the *dog* condition, dogs’ pupil size increased significantly over the course of the trial (*human outside*: *F* = 6.09, e*df* = 13.97, Ref. *df* = 15.71, *P* < 0.001; *human within*: *F* = 2.33, e*df* = 12.16, Ref. *df* = 14.19, *P* = .005). Finally, the gaze coordinates and the random term for each event contributed significantly to explain the variance in the results too (gaze coordinates: e*df* = 25.91, Ref. *df* = 28.15, *F* = 47.72, *P* < 0.001; event: e*df* = 1618.21, Ref. *df* = 1716.00, *F* = 222.57, *P* < 0.001).

#### Experiment 2.

**Figure 4.**
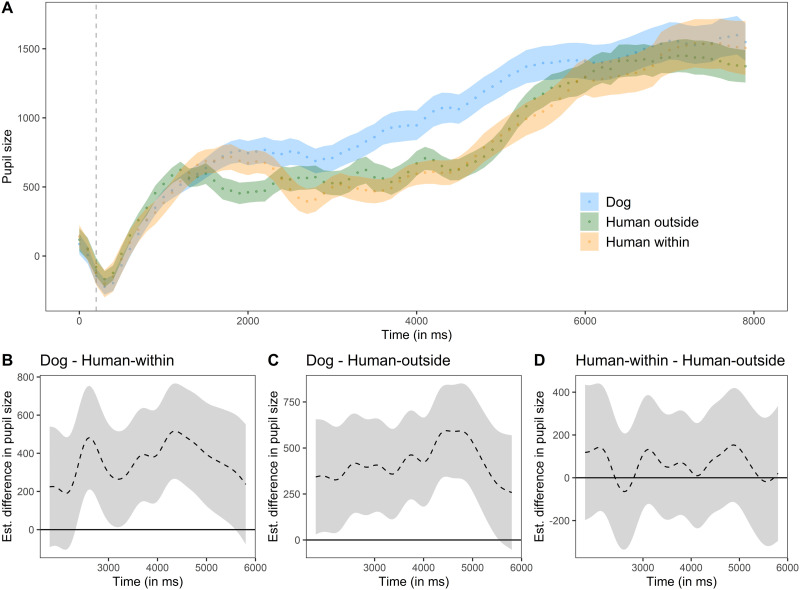
Experiment 2: A) time course of dogs’ average pupil size (arb. unit), baseline corrected and down-sampled, over the duration of the whole trial. The grey vertical dashed line indicates the end of the baseline period. The coloured dotted lines show, for each condition, the average pupil size (each dot corresponds to the average of each bin) ± standard error. B), C) and D): difference curves between conditions based on GAMM predictions. Data are plotted as estimated difference in pupil size (dashed black line) ± 95% confidence intervals (grey area).

The pre-processed pupil size across the three conditions is plotted in Figure 4A. The full model explained the results significantly better than a null model lacking the condition factor and the non-parametric regression lines of the condition levels over time (*χ*^2^ = 31.79, *df* = 8, *P* < 0.001; AIC difference: 90.07; Table S11). Dogs exhibited larger pupil size when watching the conspecific than when watching the human agent (comparison *human outside* − *dog: t* = −2.84, *P* = .005; comparison *human within* − *dog*: *t* = −2.27, *P* = .023). The difference curves (Figure 4B–D) show that there was no significant difference in pupil size between the two conditions in which the agent was human. We also found an increase in pupil size over the course of time in the *dog* (*F* = 2.03, e*df* = 10.68, Ref. *df* = 12.79, *P* = .013) and *human outside* conditions (*F* = 5.02, e*df* = 13.80, Ref. *df* = 15.67, *P* < 0.001) but not in the *human within* condition (e*df* = 8.82, Ref. *df* = 10.75, *F* = 1.66, *P* = .069). Finally, the gaze coordinates and the random term for each event contributed significantly to explain the variance in the results too (gaze coordinates: e*df* = 25.49, Ref. *df* = 28.01, F = 58.31, *P* < 0.001; event: e*df* = 1620.21, Ref. *df* = 1698.00, *F* = 308.98, *P* < 0.001).

## DISCUSSION

This study aimed at investigating the cognitive processes underlying action prediction in dogs. In particular, we intended to tease apart the role of motor representations of the observed movements from that of visual familiarity with the observed movements. Contrary to our initial prediction, dogs anticipated the immediate action target of a human from the first trial, but hardly ever anticipated the target of a conspecific’s action. Moreover, dogs were faster at shifting their gaze to the target object when the agent was a human rather than a conspecific. Overall, dogs’ gaze arrival times did not significantly differ between the two human conditions, suggesting that the saliency of the (dog compared to the human) agent rather than the similarity of the observed movements to the observers’ own motor representations influenced dogs’ action prediction. This interpretation was also supported by the pupil size analysis showing increased pupil size in the *dog* compared to the two human conditions and no significant difference in the pupil size between the two human conditions in both experiments.

In Experiment 1, showing the displacement of a ball as goal-directed action, we found evidence for action prediction only in one condition—showing a human agent walking towards and kicking a ball—when analysing looks to the target that were preceded by a look to the agent in the first trial. In contrast, dogs looked at the target object reactively when the action was performed by a dog, and their gaze arrival time to the target did not differ significantly from the agent’s arrival time when the human performed the action in a dog-like manner. This result, together with longer looking times to the human moving similarly to a dog compared to the human moving normally, hints at the possibility that for some dogs the unfamiliar movements employed by the human agent were more salient and delayed the tendency to predict her action target in the first experiment.

In Experiment 2, we only found a trend towards significantly predictive looks, and only in the condition in which the human agent performed a human-like goal-directed action: dogs’ gaze landed in the target object AOI approximately 600 ms before the agent in the first trial in which the human walked towards and grasped a toy with her fingers (see Supplementary Materials for more detailed results). We did not expect differing results between the two experiments. Given the high similarity between the scenes of the two experiments and since Experiment 2 was mostly presented on the same day after Experiment 1, it is possible that dogs’ scan path was influenced by the repetitiveness of the situation which made the stimuli of Experiment 2 less engaging. This interpretation is additionally supported by the dogs’ looking times to the agents and probability to look at least once at the target decreasing with increasing trial number in both experiments and their gaze arrival times in the target AOI becoming slower with increasing trial number in Experiment 2. Another possibility is that the events of Experiment 2, where the agents reached the target AOI sooner than in Experiment 1, did not allow enough time for the dogs to look at the agent and still exhibit predictive gaze shifts to the target afterwards, given dogs’ relatively long fixations (Park et al., 2020). Finally, limited to the dog condition, differences in the actor’s body language (more playful in Experiment 2 than 1) could have potentially led the subjects to gaze sooner at an object that is the target of play. Additional research is needed to test these post-hoc hypotheses. For these reasons, we decided to explore the data of the second experiment including also looks to the target AOI that were not preceded by looks to the agent AOI.

Prior to collecting the data, we had decided to include in the gaze arrival times analyses only trials in which dogs had gazed at the agent before gazing at the target object because we thought this was necessary to ensure that dogs were basing their predictions on the presented condition. However, the exploratory arrival times analysis of all four trials of the second experiment revealed that dogs’ gaze arrived at the target significantly sooner than the agent in the two human conditions but not in the dog condition. It is important to note that, from their viewing distance, dogs could always perceive the whole screen even if their gaze coordinates were not intersecting the agents’ AOIs. This, together with memory of the previous trials, might explain why dogs’ anticipatory looks to the target were still modulated by the condition despite the fact that dogs did not look always into the agent AOI before looking at the target. Moreover, in the first trial of Experiment 2, dogs’ gaze arrived at the target predictively in all three conditions. It is possible that the novelty of the target object (while the agents and the setting remained the same as in the previous experiment) captured the dogs’ attention and led to predictive looks to the target in all conditions on the first trial of Experiment 2. Therefore, also the results of Experiment 2 seem to highlight the importance of visual familiarity over that of the observer’s own movement repertoire, for action prediction. However, these results were not confirmed by our preregistered analyses (including only trials in which dogs looked at the agent first), that found only limited support for action prediction (only a tendency to predict in the first trial when the human agent moved normally) and no difference in the gaze arrival times across conditions.

The fact that dogs made predictive gaze-shifts to the target object of a human agent performing movements outside of their own motor repertoire suggests that, at least in dogs, first-person experience performing a certain action is not crucial for anticipating the target of that action, unlike what has been suggested for children (e.g., Hunnius & Bekkering, 2014). Under certain conditions, humans too are known for attributing goals to observed actions that fall outside of their motor repertoire (Gazzola, Rizzolatti, et al., 2007; Gazzola, van der Worp, et al., 2007; Klein et al., 2009; Vannuscorps & Caramazza, 2017). Dogs’ action prediction (in our case, the expectation that an agent will approach a salient object) does not seem to be underpinned by the observers’ movement representations of similar actions. We suggest that, at least in dogs, the role of visual experience and agent saliency might outweigh that of motor representations derived from self-produced actions, in mediating action prediction. To disentangle further the contribution of agent saliency and visual familiarity with an action, more research is needed. First-person motor experience with the observed movements was neither necessary nor sufficient for action prediction, as indicated by the fact that dogs, on average, did not anticipate the goal object of a conspecific. As a group, they only did so on the first trial of Experiment 2 (when including target looks without a preceding agent look). Different possibilities exist to explain this phenomenon. First, the majority of the tested subjects (with one exception) had already experience with watching videos of humans but not of dogs, due to their participation in previous eye-tracking experiments. A second possibility is that the dogs avoided to look at the target of the conspecific’s gaze, similarly to what was found in a study in which dogs avoided to choose the food looked at by video-projected conspecifics (Bálint et al., 2015). Third, the conspecific was likely more salient than the human agent, hence it might have been more difficult for the dogs to disengage from the conspecific than from the human agent. Lastly, limited to the first experiment, it could be hypothesized that the effector used on the ball might have been perceived as unusual (as most dogs would have typically used their muzzle in the same situation) and that this might explain why the dog agent attracted more attention than the humans. However, our results do not reconcile well with this hypothesis. Indeed, dogs’ gaze arrived to the ball reactively already during the first trial. Hence, already before having seen which effector the dog was going to use, the subjects’ gaze had already been captured by the conspecific rather than disengaging from it and landing on the target (as instead was the case in the *human outside* condition). Second, taking all four trials into account, the dogs could disengage faster from the human moving in an unusual way than from the dog agent. Hence, one would need to hypothesize that dogs’ attention is attracted only by unusual actions of conspecifics but not by unusual actions of humans. Third, in both experiments, we found that dogs looked longer and showed greater pupil size in the *dog* condition compared to the two human conditions, even if in the second experiment the dog uses a more “usual” effector.

We interpret anticipatory target looks as evidence for action prediction. Even though this is in line with previous literature (e.g., Brandone et al., 2014; Falck-Ytter et al., 2006; Myowa-Yamakoshi et al., 2012), not every look to the target object might constitute action prediction. Some might also be part of the dogs’ visual exploration of the scene. We tried to be more stringent by focusing on target looks that followed looks to the agent (with the exception of the exploratory analysis of Experiment 2, when the dogs had already had experience with a similar sequence and scene). Nevertheless, stimulus competition (Desimone & Duncan, 1995; Duncan et al., 1997) due to the conspecific being more salient than the human agent might explain the difference between the dog condition and the human conditions without necessarily appealing to action prediction. The pupillometry and looking time results seem to support the hypothesis that dogs found the conspecific more salient that the human agent. Indeed, while previous studies had already shown that dogs are able to discriminate between conspecifics and humans based on visual information alone (Autier-Dérian et al., 2013) and that dogs prefer (i.e., look longer at) static pictures of conspecifics over those of humans (Somppi et al., 2012, 2014; Törnqvist et al., 2015), we additionally provide evidence from the pupil dilation data that seeing a conspecific results in increased arousal or in an increased orienting response compared to seeing a human. Looking times and pupil dilation responses have both been considered as indices of cognitive processing of perceptually unfamiliar, salient or surprising stimuli (Eckstein et al., 2017; Jackson & Sirois, 2009). In both experiments, the looking times and pupil dilation responses yielded convergent evidence that the conspecific was more salient than the human agent. The reason for this difference is not clear. Six of the dogs in our sample lived with at least another dog (Table S1) and, although we cannot quantify our dogs’ amount of interaction with conspecifics prior to being tested, it is safe to assume that they all had visual experience with other dogs, since they lived in a large urban environment. However, it is not unreasonable to speculate that some of them had perhaps less interactions with conspecifics than with humans.

As we had preregistered, finding that dogs would predict the target object in all three conditions of these experiments could be consistent with the emulative action reconstruction account. However, we only found evidence for dogs predicting their conspecific’s goal in the first trial of Experiment 2, when including also looks at the target that were not preceded by looks at the agent, probably due to the increased saliency of the conspecific agent.

Both experiments showed videos of the same dog and human agents and our sample size was restricted by the number of dogs that could be trained for accurate eye tracking within the timeframe of the project. These factors might limit the generalizability of our results. Moreover, within each experiment, we only showed a simplified scene with only one salient goal object, always situated on the same side of the scene, to increase the chances to observe action prediction. This strategy was also used in previous experiments (e.g., Myowa-Yamakoshi et al., 2012, 2015). However, future research should investigate if dogs predict the actions of humans also in a situation in which the agent can choose among multiple target objects.

Humans’ face scanning patterns, unlike those of chimpanzees, seem to depend on the agent’s goal and context of action (Hirata & Myowa, 2018; Myowa-Yamakoshi et al., 2012, 2015). In both our experiments, dogs’ standardised looking times to the dog’s face were longer than the looking times to the body. In the first experiment, dogs’ standardised looking times at all the agents’ faces were longer than their looking times at the agents’ bodies, irrespective of condition. In the second experiment, dogs looked longer at their conspecific’s face than at each of the other agents’ AOIs (but see Supplementary text and Figure S3 and S4 for the absolute looking times). The distance between the agent’s face and the target object, however, did not seem to affect the gaze arrival times. For example, in the first trial of the first experiment, dogs’ gaze arrived to the target sooner in the condition in which the human walked normally (hence her face was more distant from the target) than in the other two conditions.

In the first experiment, dogs’ looking times were modulated by condition, with dogs looking longer at their conspecific than at the human agent moving similarly to a dog and longer at the latter than at the human agent moving normally. This pattern suggests that dogs discriminated between the dog and human agent despite the similarity of the moving pattern. Moreover, the videos showing a human moving in an unusual way caught their attention more than the video showing a human moving normally to reach the target, a difference that was only evident from the looking times but not from the pupil dilation response. In Experiment 2, dogs did not seem to differentiate with their looking times nor pupillary response between the two types (usual and unusual) of human actions. Additional experiments are needed to clarify whether this was due to a carry-over effect between experiments. However, dogs were more likely to look at the target in the *human within* compared to the other two conditions. We interpret this finding as evidence that the agent moving in an unusual way caught the dogs’ attention more and hence increased the probability that dogs looked at the target at least once during the trial.

In conclusion, our study shows that dogs can look predictively to the immediate target of a human agent’s action, despite lacking effector-specific motor representations of the observed movements. However, we cannot conclusively rule out that effects of stimulus competition rather than differences in action prediction caused the differences in arrival times between conditions. In any event, dogs’ looking to the agent’s target in our study does not seem to be automatic as dogs hardly ever predicted a conspecific’s target in the same setting in which they predicted a human’s target. Hence, our study highlights the contribution of visual familiarity and saliency of the agent over that of the observer’s motor repertoire for action prediction.

## ACKNOWLEDGMENTS

We wish to thank: the dogs and their owners; Laura Laussegger, Marion Umek and Sabrina Karl for training the dogs and collecting the data; Karin Bayer for administrative support.

## FUNDING INFORMATION

This study was funded by the Vienna Science and Technology Fund (WWTF) [10.47379/CS18012], the City of Vienna and Ithuba Capital AG through project CS18-012 and the Austrian Science Fund (FWF) through project W1262-B29.

## AUTHOR CONTRIBUTIONS

Lucrezia Lonardo: Conceptualization; Data curation; Formal analysis; Methodology; Project administration; Software; Visualization; Writing—Original draft; Writing—Review & editing. Christoph J. Völter: Conceptualization; Data curation; Formal analysis; Methodology; Software; Supervision; Visualization; Writing—Review & editing. Claus Lamm: Conceptualization; Funding acquisition; Methodology; Supervision; Writing—Review & editing. Ludwig Huber: Conceptualization; Funding acquisition; Methodology; Resources; Supervision; Writing—Review & editing. All authors agree to be accountable for the content of the work.

## DATA AVAILABILITY STATEMENT

The datasets generated and the R scripts used for this study can be found in the following Github repository: https://github.com/lonardol/action_prediction_dogs.
