# Supplementary material for: Dogs Rely On Visual Cues Rather Than On Effector-Specific Movement Representations to Predict Human Action Targets
Source: Open Mind (Camb). 2023 Aug 20;7:588–607. doi: 10.1162/opmi_a_00096 (PMC10575556; doi:10.1162/opmi_a_00096)
Supplement: Supplementary file 1 [file opmi-07-588-s001.docx]

Supplementary text

## Statistical analyses

### Gaze arrival times

For both experiments, following our preregistered plans, we initially fitted a Linear Mixed Model (LMM; Baayen, 2008) to analyse the effect of condition on dogs’ standardised gaze arrival times into the target IA. We checked the assumptions of these models using Shapiro-Wilk normality tests and inspecting plots of residuals against fitted values (Queen & Keough, 2002). The assumptions were not met (for Experiment 1: W = 0.952, P <0.001; for Experiment 2: W = 0.967, P < 0.001).

In the beta models, the correlation between random intercept and slopes was not included. The covariates were z-transformed prior to entering the model (Schielzeth, 2010) and the random slope of condition was manually dummy coded and centered to ensure that the results were not dependent on the reference category of condition. We used the R function “drop1” of package “lme4” (Bates et al., 2015) to draw inference about the fixed effects. We verified that the models were not overdispersed by running an overdispersion test (Experiment 1 model with all trials: χ^2^ = 125.47, df = 160, P = 0.980, dispersion parameter = 0.784; Experiment 2 model with all trials: χ^2^ = 139.64, df =162, P = 0.898, dispersion parameter = 0.862; Experiment 1 model with first trial only: χ^2^ = 30.92, df = 39, P =0.819, dispersion parameter = 0.793; Experiment 2 model with first trial only: χ^2^ = 37.021, df =40, P = 0.605, dispersion parameter = 0.926) and we assessed the distribution of the best linear unbiased predictors (BLUPs; Baayen, 2008)). The stability of the estimated coefficients with regards to possible influential cases was evaluated by excluding the levels of the random effects one at a time (Nieuwenhuis, 2012). For Experiment 1, this revealed both models to be stable. For Experiment 2, the model with all trials was of acceptable stability, with the exception of the effects of condition – human outside and order of presentation of the conditions, and the model with the first trial only was of acceptable stability. Ninety-five% confidence intervals for model estimates were obtained by means of parametric boostrap (1000 repetitions).

### Looking times

Because visual inspection of the distribution of the absolute and standardized looking times revealed that the response was not symmetric, analyses were conducted using the square-root of the original looking times as response.

To analyse the effect of condition (dog/human within/human outside), IA (agent’s face/body) and their interaction (fixed effects) on our subjects’ looking times to the agents (square root-transformed), we fitted a LMM (LMM; Baayen, 2008) using the function “lmer” of package “lme4” (Bates et al., 2015). We included in this full model the interaction between the two test predictors because dogs’ might have allocated their attention to the moving agents’ face or body differently across agents’ species (conspecific vs heterospecific) and type of movements (within or outside their own motor repertoire). Additional fixed effects were the trial number (1 to 4) and the order of conditions (1 to 3). Both covariates were z-transformed prior to entering the model to achieve a more easily interpretable model output (Schielzeth, 2010). The levels of condition and IA were manually dummy coded and centered for the random effects part of the model. Finally, we included the random slope of the interaction between condition and IA and the random slopes of trial number and order of presentation of the conditions within the random intercept of subject. The correlations between random slopes and intercept were not included. Whenever necessary (i.e., for the standardised looking times of both experiments), prior to fitting beta models, we transformed the response such that it did not comprise the exact values of zero and one (Smithson & Verkuilen, 2006).

For both experiments, the residuals resulted normally distributed for the absolute looking times (Shapiro-Wilk test - Experiment1: W = 0.993, P = 0.061; Experiment2: W = 0.995, P = 0.185) but not for the standardized looking times (Experiment1: W = 0.969, P < 0.001; Experiment2: W = 0.978, P < 0.001). Therefore, since the response was in both cases comprised between zero and one, we fitted a GLMM with beta error structure and logit link function. The fixed and random effects parts were identical to those of the previously described LMM.

We verified the assumptions and stability of the models as described above. For Experiment 1, both models (absolute and standardised looking times) were stable. The standardised looking times model was not overdispersed (χ^2^ = 376.69, df = 369, P = 0.380, dispersion parameter = 1.021). For Experiment 2, both models were of acceptable stability and the standardised looking times model was not overdispersed (χ^2^ = 351.43, df = 367, P = 0.712, dispersion parameter = 0.958).

As an overall test of the interaction between condition and IA, using a likelihood ratio test, we compared the full model to a reduced model lacking the interaction but retaining the fixed effects of IA and condition and all other fixed and random effects of the full model.

For Experiment 1, the effect of the interaction in the model fitted to the absolute (χ^2^ = 3.80, df =2, P = 0.149) and standardised looking times (χ^2^ = 4.81, df = 2, P = 0.091) was not significant. Therefore, further analysis was conducted using the respective reduced models, lacking the fixed effect of the interaction but retaining the fixed effect of both predictors and all other random and fixed effects of the full model described above.

For both experiments, to avoid overconfidence in our results (Schielzeth & Forstmeier, 2009) we compared the fit of the full models with those of null models using a likelihood ratio test. The null models lacked both the fixed effects and the interaction of condition and IA but were otherwise identical to the full models.

To draw inference about the single fixed effects, Ps were calculated with Satterthwaite approximation for LMMs based on restricted maximum likelihood (Luke, 2017), using the function “lmer” of package “LmerTest” (Kuznetsova et al., 2017). The overall significance of the fixed effects of the standardised looking times beta models was tested using the function “drop1”. To run a comparison between the non-reference levels of condition, we used, on LMMs fitted with restricted maximum likelihood, the function “glht” of package “multcomp” (Hothorn et al., 2008), which runs simultaneous tests for general linear hypotheses and corrects the Ps using Tukey’s method. For the standardised looking times beta models, pairwise comparisons of the average looking times between conditions and IAs were obtained using the function “lsmeans” of the homonymous package (Lenth, 2016), which employs t-tests and corrects the Ps for multiple comparisons using Tukey’s method.

### Pupil size

We removed from the raw data artifacts related to blink events, by removing samples from 100 ms before each blink to 100 ms after it. Resulting gaps of up to 500 ms were filled by means of linear interpolation. We only included in the analyses trials in which dogs looked on-screen for at least 70% of the whole trial duration. This criterion resulted in the exclusion of one trial from the “human outside” condition of Experiment 1 and three trials, all from the “human within” condition, of Experiment 2. We performed a subtractive baseline correction to account for individual variations in pupil size across subjects and trials. Namely, for each subject and trial, we subtracted from the pupil size in every ms after the end of the baseline period, the median pupil size for that subject and trial. We chose the first 200 ms of stimulus presentation as baseline period because during this period the agents had not yet started to move. To reduce autocorrelation due to the relatively slow physiological response times, we down-sampled the data to the median value of each 100-ms bin. As in previous studies (e.g., Völter & Huber, 2021), for both experiments we chose a 4-s response window (from 3000 to 7000 ms).

For each experiment, we compared the minimized smoothing parameter selection score of this model to that of a null model lacking the condition factor and the non-parametric regression lines of the condition levels over time, by applying a χ^2^ test on the difference in scores and the difference in degrees of freedom. To this end, we used the function “compareML” of package “itsadug” (Van Rij et al., 2015). Additionally, to evaluate the significance of condition, we visually inspected the estimated differences between conditions using the function “plot_diff” of package “itsadug”.

We assessed autocorrelation by visually inspecting the correlations between the residuals and the lagged residuals (function “acf” of package “stats”; R Core Team (2021)). We checked the normality and homogeneity of the distribution of the residuals by inspecting a QQ-plot and a plot of residuals against fitted values (function “gam.check” of package “mgcv”). These checks verified that the autocorrelation was not excessive and that the assumptions of the model were met.

**Results**

**Gaze arrival times**

**Experiment 1**

The average standardized gaze arrival time in the dog condition was 291.68 ms (t = 1.37, df = 15, P = 0.190), in the human outside condition -273.45 ms (t = -1.25, df = 15, P = 0.232) and in the human within condition - 490.17 ms (t = -1.26, df = 15, P = 0.228). As shown in Figure 1A, there was no difference in the average standardized gaze arrival times between the two human conditions (comparison human outside – human within: t = 0.62, df = 15, P = 0.543) and between the dog and human outside condition (t =1.87, df =15, P =0.081).

**Experiment 2**

The average standardized gaze arrival time in the dog condition was -217.67 ms (t = -1.02, df = 13, P = 0.327), in the human outside condition -200.62 ms (t = -0.95, df = 15, P = 0.360) and in the human within condition -241.55 ms (t = -0.89, df = 15, P = 0.388). There was no difference in the average standardized gaze arrival times between conditions (paired-sample t-test comparing the conditions dog-human outside: t = 0.36, df = 13, P = 0.7286; comparison dog-human within: t = 0.73, df = 13, P = 0.481; comparison human outside-human within: t = -0.22, df = 15, P = 0.833).

The average standardized gaze arrival time in the first trial of the dog condition was -451.39 ms (t = -1.41, df = 13, P = 0.1812), in the human outside condition -608.91 ms (t = -1.91, df = 15, P = 0.075) and in the human within condition -467.1 ms (t = -1.27, df = 15, P = 0.223). There was no difference between conditions in the average standardized gaze arrival times on the first trial (paired-sample t-test comparing the conditions dog-human outside: t = 0.64, df = 13, P = 0.534; comparison dog-human within: t = 0.355, df = 13, P = 0.729; comparison human outside-human within: t = -0.367, df = 15, P = 0.719).

**Looking times**

**Experiment 1**

When analysing the absolute looking times (Figure S3), the full model explained the results better than the null one (χ^2^ =16.491, df = 5, P = 0.006). As shown in Table S12, dogs looked longer at the dog agent than at the human agent moving normally (comparison human outside – dog: t = -3.94, df =16.34, P = 0.001) and they looked longer at the human moving similarly to a dog than at the human moving normally (comparison human within - human outside: z = 2.44, P = 0.038). There was no difference between the looking times to the human agent moving similarly to a dog and to the dog agent (t = -1.82, df =15.89, P = 0.484). The main effect of IA as well as that of order of presentation of the conditions were not significant. Finally, with increasing trial number, dogs looked for shorter periods of time to the agents (t = -3.36, df = 280.97, P = 0.01).

**Experiment 2**

In Experiment 2, when analysing the absolute looking times (Figure S4), the full-reduced model comparison revealed to be significant, suggesting that the interaction between IA and condition contributed to explain the variation in dogs’ looking times (χ2 =14.24, df = 2, P < 0.001).

As shown in Table S13, dogs spent more time looking at the agent’s body than at its face when the agent was a conspecific compared to when it was a human (human outside-dog*IA face: t = -2.79, df = 14.98, P = 0.014; human within-dog*IA face: t = -3.15, df = 14.98, P = 0.007). Dogs’ looking times to the agents’ face and body did not differ between the two human conditions (human outside-human within*IA face: t = -0.17, df = 15.22, P = 0.868). Dogs’ looking times to the agents decreased significantly with increasing trial number (t = -4.14, df = 287.23, P < 0.001). Post-hoc pairwise comparisons of the absolute looking times revealed that dogs looked significantly longer at their conspecific’s face than at the human’s face (dog face – human outside face: t = 4.38, df = 23.4, P = 0.003; dog face – human within face: df =25.4, t = 4.46, P = 0.002). They also looked longer at their conspecifics’ body than at the human’s face (dog body – human outside face: t = 3.93, df = 46, P = 0.004; dog body – human within face: t = 3.35, df = 45.9, P = 0.019). Finally, they looked longer at the human’s body than face (human outside body – human outside face: t = 3.19, df =33.5, P = 0.033; human outside body – human within face: t = 4.514, df =37.9, P = 0.001; human within body – human outside face: t = 5.31, df = 37.9, P < 0.001; human within body – human within face: t = 3.30, df = 40.3, P = 0.024).

Tables

Table S1

Demographic information of the 16 tested dogs

| Dog ID | Sex | Neutered | Age (Months) | Breed | 1^st^ Condition - Ball | 1^st^ Condition - Elephant | Only dog in the household |
| --- | --- | --- | --- | --- | --- | --- | --- |
| 01 | M | Yes | 28 | Mix | Human outside | Dog | Yes |
| 02 | M | No | 29 | Collie (rough) | Human within | Human within | Yes |
| 03 | F | Yes | 45 | Labrador retriever | Dog | Dog | Yes |
| 04 | M | Yes | 31 | Mix | Dog | Dog | No – 1 more |
| 05 | F | Yes | 37 | Labrador retriever | Human within | Human outside | No – 3 more |
| 06 | F | No | 32 | Mix | Human outside | Human within | No – 1 more |
| 07 | F | Yes | 48 | Mix | Dog | Dog | Yes |
| 08 | M | Yes | 70 | Mix | Dog | Human outside | Yes |
| 09 | F | No | 35 | Border Collie | Human within | Human outside | Yes |
| 10 | M | No | 60 | Australian Shepherd | Human within | Human outside | Yes |
| 11 | M | Yes | 20 | Kleiner Münsterländer | Human outside | Human within | Yes |
| 12 | M | Yes | 87 | Canarian Warren Hound | Human within | Dog | No – 2 more |
| 13 | F | No | 139 | Border Collie | Human outside | Human within | Yes |
| 14 | M | Yes | 30 | Border Collie | Human outside | Human within | No – 2 more |
| 15 | M | Yes | 84 | Mix | Human outside | Human outside | Yes |
| 16 | M | Yes | 61 | Australian Shepherd | Dog | Human within | No – 1 more |

Table S2

Experiment 1: results of the beta GLMM investigating the effect of condition on the proportion of trial time elapsed before dogs looked at the target IA.

| Term | Estimate | Std. Error | χ^2^ | Df | CI min | CI max | P | Min | Max |
| --- | --- | --- | --- | --- | --- | --- | --- | --- | --- |
| Intercept | 0.56 | 0.15 |  |  | 0.26 | 0.85 | ^(1)^ | 0.47 | 0.61 |
| Condition – human outside^(2)^ | -0.32 | 0.17 |  |  | -0.65 | 0.01 |  | -0.42 | -0.22 |
| Condition – human within^(2)^ | -0.46 | 0.17 | 8.06 | 2 | -0.79 | -0.13 | 0.018 | -0.51 | -0.35 |
| Trial^(3)^ | 0.04 | 0.05 | 0.62 | 1 | -0.06 | 0.15 | 0.430 | -0.01 | 0.06 |
| Order of conditions^(3)^ | 0.03 | 0.07 | 0.14 | 1 | -0.11 | 0.16 | 0.713 | -0.02 | 0.06 |

*Note*:

(1) Not indicated due to its limited interpretability

(2) Reference category: dog. The indicated test refers to the overall effect of condition

(3) Z-transformed

Table S3

Experiment 1: results of the beta GLMM investigating the effect of condition on the proportion of trial time elapsed before dogs looked at the target IA in the first trial only

| Term | Estimate | Std. Error | χ^2^ | Df | CI min | CI max | P | Min | Max |
| --- | --- | --- | --- | --- | --- | --- | --- | --- | --- |
| Intercept | 0.79 | 0.23 |  |  | 0.39 | 1.29 | ^(1)^ | 0.65 | 0.84 |
| Condition -human outside^(2)^ | -1.04 | 0.26 |  |  | -1.55 | -0.55 |  | -1.13 | -0.94 |
| Condition -human within^(2)^ | -0.65 | 0.25 | 14.17 | 2 | -1.19 | -0.19 | 0.001 | -0.79 | -0.49 |
| Order of conditions^(3)^ | 0.04 | 0.11 | 0.14 | 1 | -0.17 | 0.25 | 0.708 | -0.01 | 0.08 |

*Note*:

(1) not reported due to its very limited interpretability

(2) comparison with the reference level: dog; the indicated test refers to the overall effect of condition

(3) z-transformed to a mean of 0 and standard deviation of 1

Table S4

Experiment 2: results of the beta GLMM investigating the effect of condition on the proportion of trial time elapsed before dogs looked at the target IA.

| Term | Estimate | Std. Error | χ^2^ | Df | CI min | CI max | P | Min | Max |
| --- | --- | --- | --- | --- | --- | --- | --- | --- | --- |
| Intercept | -0.48 | 0.14 |  |  | -0.75 | -0.19 | ^(1)^ | -0.52 | -0.39 |
| Condition -human outside^(2)^ | 0.00 | 0.15 |  |  | -0.29 | 0.29 |  | -0.12 | 0.07 |
| Condition -human within^(2)^ | -0.18 | 0.13 | 2.09 | 2 | -0.45 | 0.08 | 0.353 | -0.26 | -0.07 |
| Trial ^(3)^ | 0.14 | 0.04 | 7.86 | 1 | 0.06 | 0.21 | 0.005 | 0.11 | 0.15 |
| Order of conditions^(3)^ | -0.00 | 0.08 | 0.00 | 1 | -0.17 | 0.15 | 0.976 | -0.07 | 0.05 |

(1) Not indicated due to its limited interpretability

(2) Reference category: dog. The indicated test refers to the overall effect of condition

(3) Z-transformed

Table S5

Experiment 2: results of the beta GLMM investigating the effect of condition on the proportion of trial time elapsed before dogs looked at the target IA after looking at the agent in the first trial only

| Term | Estimate | Std. Error | χ^2^ | Df | CI min | CI max | P | Min | Max |
| --- | --- | --- | --- | --- | --- | --- | --- | --- | --- |
| Intercept | -0.63 | 0.21 |  |  | -1.02 | -0.24 | ^(1)^ | -0.69 | -0.53 |
| Condition -human outside^(2)^ | -0.12 | 0.26 |  |  | -0.61 | 0.35 |  | -0.27 | 0.04 |
| Condition -human within^(2)^ | -0.07 | 0.25 | 0.22 | 2 | -0.55 | 0.40 | 0.895 | -0.20 | 0.10 |
| Order of conditions^(3)^ | 0.03 | 0.11 | 0.10 | 1 | -0.19 | 0.23 | 0.751 | -0.03 | 0.10 |

*Note*:

(1) not reported due to its very limited interpretability

(2) comparison with the reference level: dog; the indicated test refers to the overall effect of condition

(3) z-transformed to a mean of 0 and standard deviation of 1

| Term | Estimate | Std. Error | χ^2^ | Df | CI min | CI max | P | Min | Max |
| --- | --- | --- | --- | --- | --- | --- | --- | --- | --- |
| Intercept | -0.71 | 0.17 |  |  | -1.06 | -0.38 | ^(1)^ | -0.79 | -0.60 |
| Condition - Human outside ^(2)^ | -0.60 | 0.17 |  |  | -0.94 | -0.27 |  | -0.70 | -0.52 |
| Condition - Human within^(2)^ | -0.37 | 0.21 | 9.58 | 2 | -0. 77 | 0.04 | 0.008 | -0.49 | -0.27 |
| Trial^(3)^ | 0.18 | 0.07 | 6.10 | 1 | 0.05 | 0.32 | 0.014 | 0.14 | 0.21 |
| Order of conditions^(3)^ | -0.00 | 0.10 | 0.00 | 1 | -0.16 | 0.17 | 0.973 | -0.07 | 0.08 |

Table S6

Experiment 2: Results of the exploratory beta GLMM investigating the effect of condition on dogs’ gaze arrival times to the target IA, including also trials in which the dogs did not look at the agent before looking at the target.

(1) Not reported due to its limited interpretability

(2) Comparison with the reference level: dog; the indicated test refers to the overall effect of condition

(3) Z-transformed

Table S7

Experiment 2: Results of the exploratory beta GLMM investigating, for the first trial only, the effect of condition on dogs’ gaze arrival times to the target IA, including also trials in which the dogs did not look at the agent before looking at the target.

| Term | Estimate | Std. Error | χ^2^ | Df | CI min | CI max | P | Min | Max |
| --- | --- | --- | --- | --- | --- | --- | --- | --- | --- |
| Intercept | -1.12 | 0.25 |  |  | -1.66 | -0.64 |  | -1.20 | -1.02 |
| Condition - Human outside ^(2)^ | -0.33 | 0.30 |  |  | -0.90 | 0.28 |  | -0.45 | -0.21 |
| Condition - Human within^(2)^ | -0.22 | 0.29 | 1.22 | 2 | -0.80 | 0.39 | 0.543 | -0.41 | -0.10 |
| Order of conditions^(3)^ | -0.18 | 0.12 | 2.15 | 1 | -0.45 | 0.06 | 0.143 | -0.25 | -0.14 |

(1) Not reported due to its limited interpretability

(2) Comparison with the reference level: dog; the indicated test refers to the overall effect of condition

(3) Z-transformed

Table S8

Experiment 1: results of the beta GLMM investigating dogs’ standardized looking times (relative to the IA size) to the agents’ face and body

| Term | Estimate | Std. Error | χ^2^ | Df | CI min | CI max | P | Min | Max |
| --- | --- | --- | --- | --- | --- | --- | --- | --- | --- |
| Intercept | -3.34 | 0.18 |  |  | -3.72 | -3.00 | ^(1)^ | -3.40 | -3.26 |
| Condition – human outside^(2)^ | -0.85 | 0.11 |  |  | -1.07 | -0.63 |  | -0.92 | -0.78 |
| Condition – human within^(2)^ | -0.39 | 0.12 | 29.74 | 2 | -0.64 | -0.15 | < 0.001 | -0.45 | -0.33 |
| IA – face | 0.78 | 0.27 | 6.87 | 1 | 0.29 | 1.30 | 0.009 | 0.67 | 0.92 |
| Order of conditions^(3)^ | -0.04 | 0.06 | -0.64^(4)^ | ^(4)^ | -0.16 | 0.08 | 0.52^(4)^ | -0.07 | 0.01 |
| Trial^(3)^ | -0.10 | 0.03 | 8.06 | 1 | -0.17 | -0.03 | 0.005 | -0.12 | -0.08 |

*Note*:

(1) Not reported due to its very limited interpretability

(2) Comparison with the reference level: dog. The reported test refers to the overall effect of condition. The comparison between levels human outside and human within was estimated to be: -0.46 ± 0.13, t = -3.60, P = 0.001. The comparison human outside – dog: -0.85 ± 0.11, t = -7.70, P <0.001 and the comparison human within – dog: -0.39 ± 0.12, t = -3.18, P = 0.005

(3) Z-transformed to a mean of 0 and standard deviation of 1

(4) χ^2^ could not be estimated, hence a Wald test is reported.

Table S9

Experiment 2: results of the beta GLMM investigating dogs’ standardized looking times (relative to the IA size) to the agents’ face and body

| Term | Estimate | Std. Error | χ^2^ | Df | CI min | CI max | P | Min | Max |
| --- | --- | --- | --- | --- | --- | --- | --- | --- | --- |
| Intercept | -4.06 | 0.16 |  |  | -4.37 | -3.76 | ^(1)^ | -4.13 | -4.01 |
| Condition – human outside^(2)^ | -0.18 | 0.23 |  |  | -0.61 | 0.26 |  | -0.25 | -0.09 |
| Condition – human within^(2)^ | -0.20 | 0.22 |  |  | -0.63 | 0.21 |  | -0.29 | -0.11 |
| IA – face^(3)^ | 0.97 | 0.26 |  |  | 0.44 | 1.49 |  | 0.88 | 1.07 |
| Order of conditions^(4)^ | -0.08 | 0.06 | 1.34 | 1 | -0.21 | 0.05 | 0.237 | -0.12 | -0.04 |
| Trial^(4)^ | -0.10 | 0.03 | 8.84 | 1 | -0.17 | -0.05 | 0.003 | -0.12 | -0.09 |
| Condition human outside*IA face^(5)^ | -1.09 | 0.41 |  |  | -1.86 | -0.25 |  | -1.34 | -0.91 |
| Condition human within*IA face^(5)^ | -0.65 | 0.38 | 8.30 | 2 | -1.39 | 0.05 | 0.016 | -0.86 | -0.49 |

*Note*:

(1) Not reported due to its very limited interpretability

(2) Comparison with the reference level: dog. A test for the main effect is not shown due to the significance of the interaction

(3) Comparison with the reference level: body. A test for the main effect is not shown due to the significance of the interaction

(4) Z-transformed to a mean of 0 and standard deviation of 1

(5) Comparison with the reference level: dog*IA body. The reported test refers to the overall effect of the interaction

Table S10

Experiment 1: Results of the GAMM fitted to the baseline corrected pupil size data

| Parametric coefficients |  |  |  |  |
| --- | --- | --- | --- | --- |
|  | Estimate | Std. Error | t | P |
| Intercept | 994.88 | 80.71 | 12.33 | < 0.001 |
| Condition - human outside^(1)^ | -323.16 | 114.90 | -2.81 | 0.005 |
| Condition - human within^(1)^ | -341.17 | 114.06 | -2.99 | 0.003 |
| Smooth terms |  |  |  |  |
|  | edf | Ref.df | F | P |
| s(time) | 12.50 | 14.38 | 4.82 | < 0.001 |
| s(time)*Condition - dog | 1.06 | 1.07 | 1.40 | 0.230 |
| s(time)*Condition – human outside | 13.97 | 15.71 | 6.09 | < 0.001 |
| s(time)*Condition – human within | 12.16 | 14.19 | 2.33 | 0.005 |
| s(Xgaze, Ygaze) | 25.91 | 28.15 | 47.72 | < 0.001 |
| s(time, Event) | 1618.21 | 1716.00 | 222.57 | < 0.001 |

*Note*: (1) reference level of condition: dog

Table S11

Experiment 2: results of the GAMM fitted to the baseline corrected pupil size data

| Parametric coefficients |  |  |  |  |
| --- | --- | --- | --- | --- |
|  | Estimate | Std. Error | t | P |
| Intercept | 1236.01 | 90.18 | 13.706 | < 0.001 |
| Condition - human outside^(1)^ | -363.23 | 127.71 | -2.844 | 0.005 |
| Condition - human within^(1)^ | -293.32 | 129.06 | -2.273 | 0.023 |
| Smooth terms |  |  |  |  |
|  | edf | Ref.df | F | P |
| s(time) | 2.66 | 3.02 | 0.28 | 0.837 |
| s(time)*Condition - dog | 10.68 | 12.79 | 2.03 | 0.013 |
| s(time)*Condition – human outside | 13.802 | 15.670 | 5.015 | < 0.001 |
| s(time)*Condition – human within | 8.82 | 10.75 | 1.66 | 0.069 |
| s(Xgaze, Ygaze) | 25.494 | 28.011 | 58.307 | < 0.001 |
| s(time, Event) | 1620.206 | 1698.000 | 308.984 | < 0.001 |

*Note*: (1) reference level of condition: dog

Table S12

Experiment 1: results of the LMM investigating dogs’ absolute looking times (square-root transformed) to the agents’ face and body

| Term | Estimate | Std. Error | t | Df | CI min | CI max | P | Min | Max |
| --- | --- | --- | --- | --- | --- | --- | --- | --- | --- |
| Intercept | 40.87 | 3.44 | 11.50 | 37.35 | 34.40 | 47.49 | ^(1)^ | 39.72 | 42.32 |
| Condition – human outside^(2)^ | -8.74 | 2.11 | -3.94 | 16.34 | -12.93 | -4.40 | 0.001 | -10.20 | -7.55 |
| Condition – human within^(2)^ | -1.82 | 2.42 | -1.82 | 15.89 | -6.60 | 3.00 | 0.484 | -2.97 | -0.65 |
| IA – face | -5.56 | 4.98 | -1.08 | 15.00 | -15.39 | 4.25 | 0.297 | -7.64 | -3.15 |
| Order of conditions | -1.28 | 1.02 | -1.17 | 15.20 | -3.33 | 0.72 | 0.261 | -2.00 | -0.58 |
| Trial^(3)^ | -2.14 | 0.64 | -3.36 | 280.97 | -3.40 | -0.85 | 0.001 | -2.36 | -1.86 |

*Note*:

(1) Not reported due to its very limited interpretability

(2) Comparison with the reference level: dog; the pairwise comparison between the levels human within and human outside was estimated to be 6.92 ± 2.84, z = 2.44, P = 0.038

(3) Z-transformed to a mean of 0 and standard deviation of 1

Table S13

Experiment 2: results of the LMM investigating dogs’ absolute looking times (square-root transformed) to the agents’ face and body

| Term | Estimate | Std. Error | t | Df | CI min | CI max | P | Min | Max |
| --- | --- | --- | --- | --- | --- | --- | --- | --- | --- |
| Intercept | 31.85 | 2.54 | 12.17 | 70.72 | 26.88 | 36.71 | ^(1)^ | 30.70 | 32.83 |
| Condition - Human outside^(2)^ | 2.87 | 3.76 | 0.73 | 20.12 | -4.65 | 10.60 | 0.472 | 1.15 | 5.52 |
| Condition - Human within^(2)^ | 3.95 | 3.28 | 1. 31 | 22.67 | -2.28 | 10.23 | 0.269 | 2.21 | 5.43 |
| IA - face | 1.33 | 4.20 | 0.31 | 42.65 | -6.97 | 9.46 | 0.760 | -0.00 | 3.11 |
| Order of conditions^(3)^ | -0.92 | 0.99 | -0.89 | 12.61 | -2.97 | 0.95 | 0.393 | -1.53 | -0.46 |
| Trial^(3)^ | -1.94 | 0.47 | -4.14 | 287.23 | -2.81 | -1.03 | <0.001 | -2.16 | -1.67 |
| Condition human outside*IA face^(4)^ | -20.04 | 6.93 | -2.79 | 14.98 | -33.83 | -6.46 | 0.014 | -24.86 | -17.28 |
| Condition human within*IA face^(4)^ | -19.23 | 5.89 | -3.15 | 14.98 | -30.38 | -7.59 | 0.007 | -22.56 | -16.74 |

*Note*:

(1) Not reported due to its very limited interpretability

(2) Comparison with the reference level: dog. The comparison between conditions human within and human outside was estimated to be: 0.99 ± 4.82, z = 0.21, P = 0.976

(3) Z-transformed to a mean of 0 and standard deviation of 1

(4) Comparison with the reference level: dog*IA body. The comparison between conditions human outside*IA face and human within*IA face was estimated to be: -0.81 ± 4.63, t = -0.17, df =15.22, P = 0.868

Figures


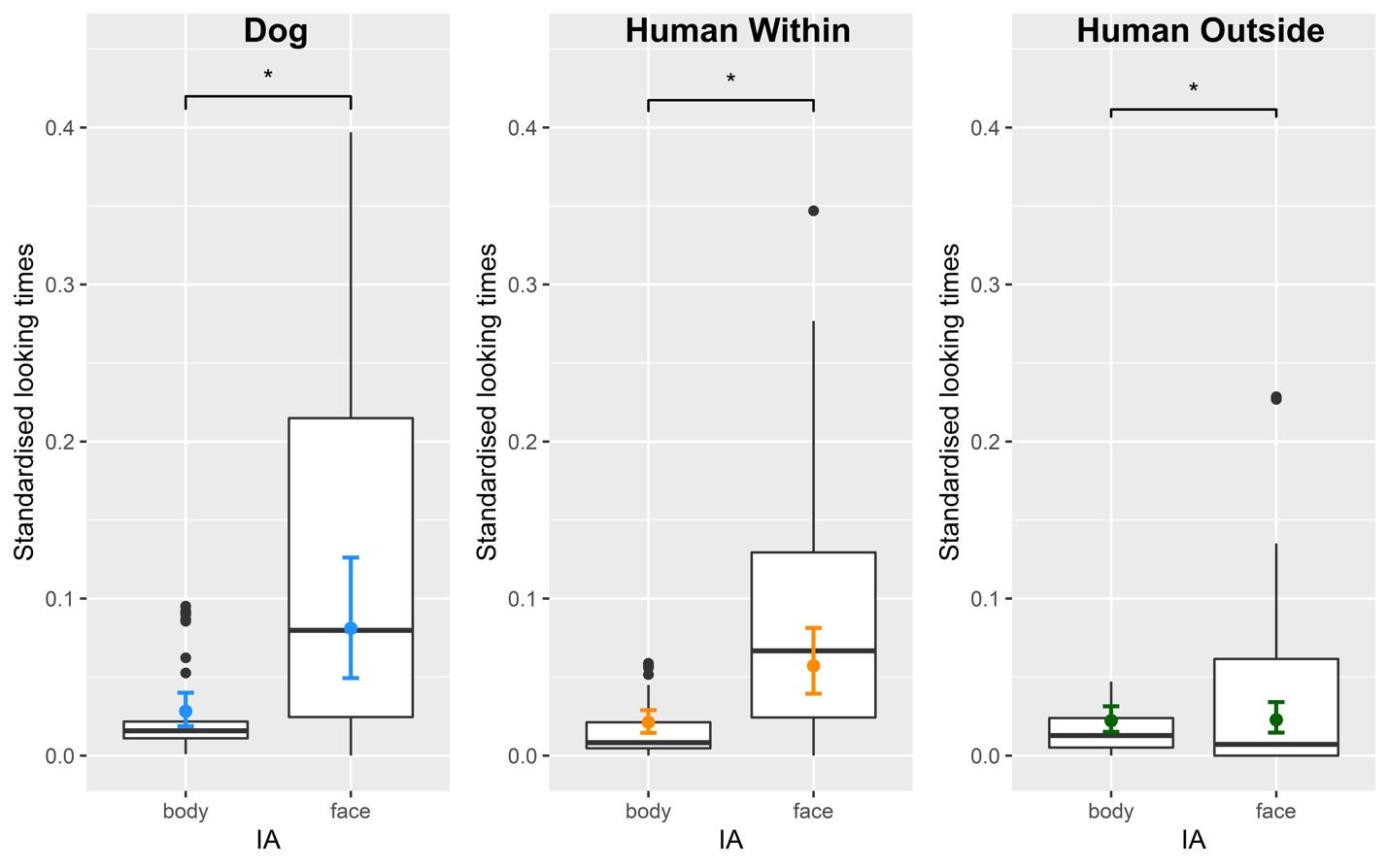
*Figure S1*. Experiment 1: looking times to the agents standardised by the size of the IA. The coloured dots and error bars represent the fitted values and the confidence intervals of the beta models, respectively

*Figure S2*. Experiment 2: standardised looking times to the agents, subdivided by condition and interest area (IA). The coloured dots and error bars represent the fitted values and the confidence intervals of the beta models, respectively.
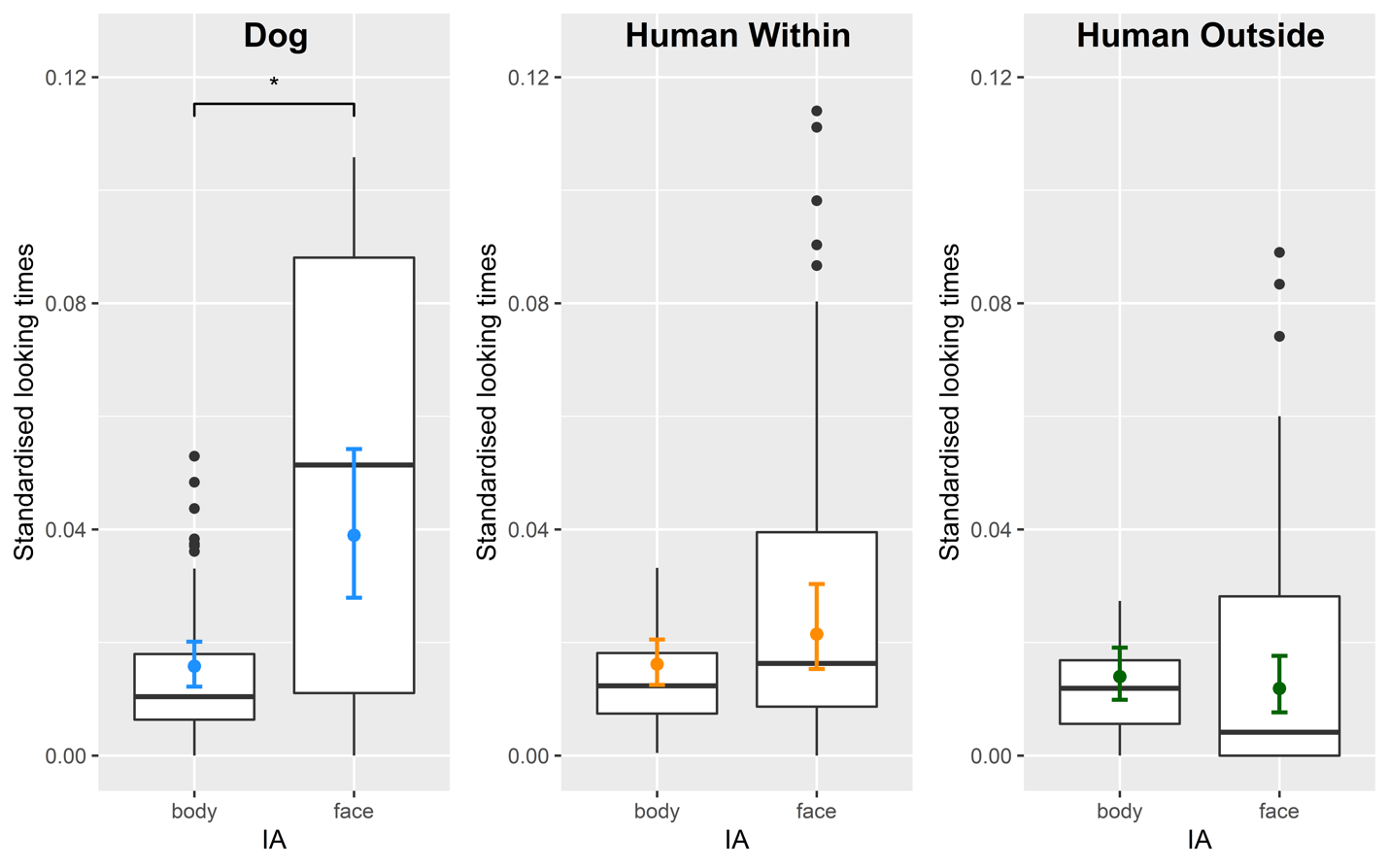


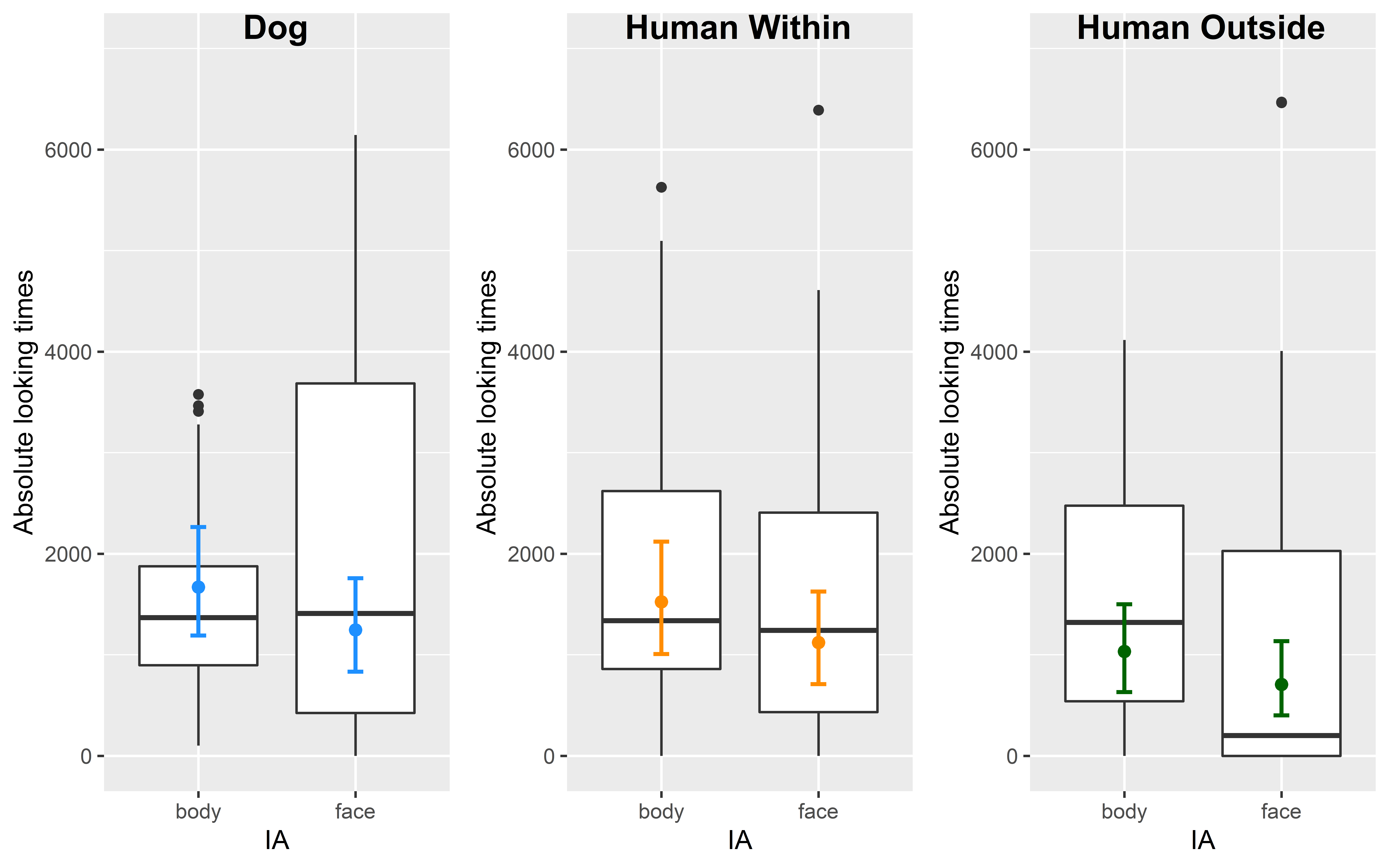
F

*Figure S3*. Experiment 1: Absolute looking times to the agents (ms), subdivided by condition and interest area (IA). The coloured dots and error bars represent the fitted values and the confidence intervals of the beta model, respectively.


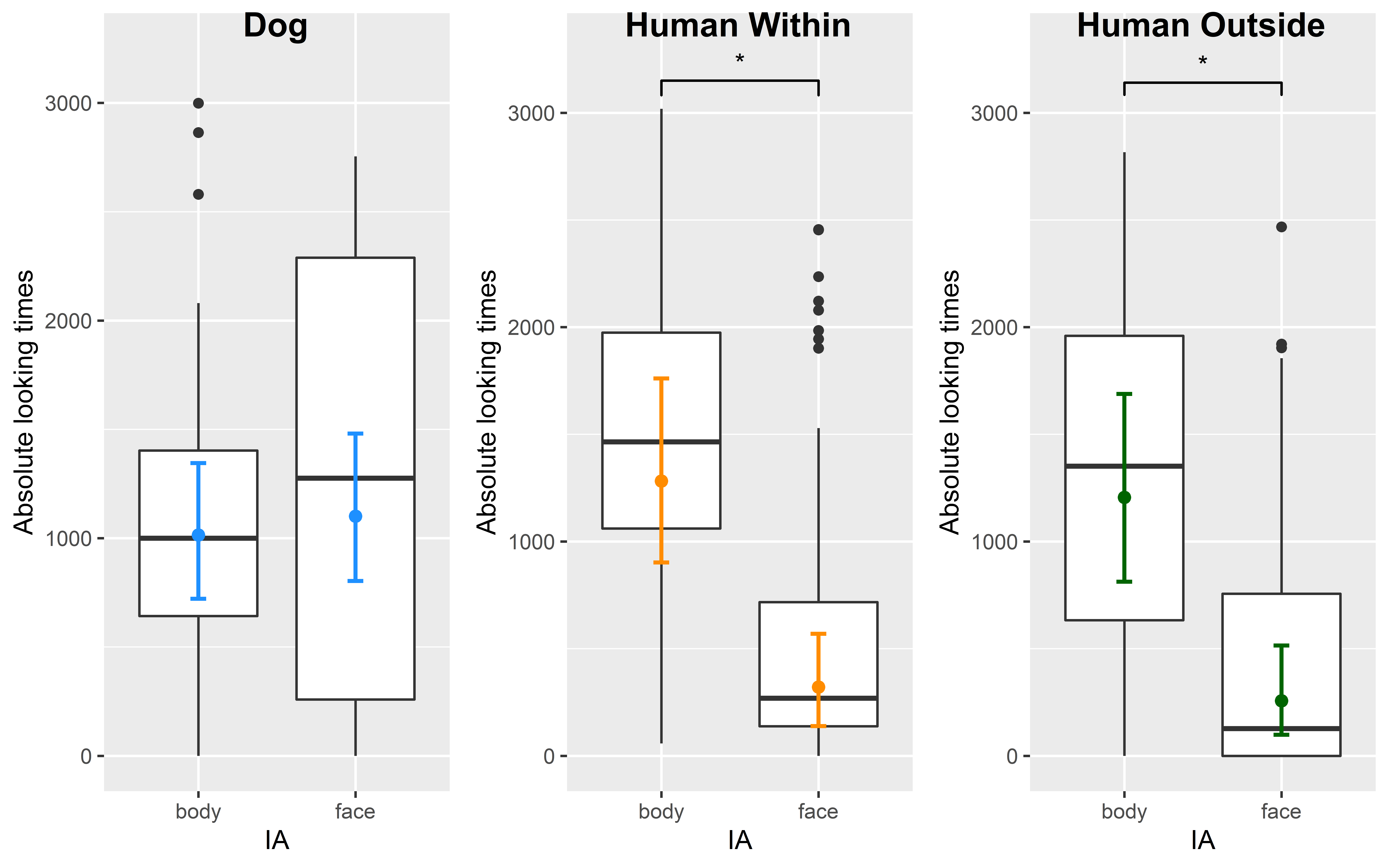


Figure S4. Experiment 2: Absolute looking times to the agents (ms), subdivided by condition and interest area (IA). The coloured dots and error bars represent the fitted values and the confidence intervals of the beta model, respectively.
